# Regulation of Thyroid Hormone-, Oestrogen- and Androgen-Related Genes by Triiodothyronine in the Brain of *Silurana tropicalis*

**DOI:** 10.1111/j.1365-2826.2010.02047.x

**Published:** 2010-09

**Authors:** Paula Duarte-Guterman, Vance L Trudeau

**Affiliations:** Centre for Advanced Research in Environmental Genomics, Department of Biology, University of OttawaOttawa, Ontario, Canada

**Keywords:** *Xenopus tropicalis*, metamorphosis, receptor, aromatase, 5α-reductase, deiodinases

## Abstract

Amphibian metamorphosis is an excellent example of hormone-dependent control of development. Thyroid hormones (THs) regulate almost all aspects of metamorphosis, including brain development and larval neuroendocrine function. Sex steroids are also important for early brain function, although little is known about interactions between the two hormonal systems. In the present study, we established brain developmental profiles for thyroid hormone receptors (*tralpha* and *trbeta*), deiodinases (*dio1*, *dio2* and *dio3*), aromatase (cyp19) mRNA and activity, oestrogen receptors (*eralpha* and *erbeta*), androgen receptor (*ar*) and 5α-reductases (*srd5alpha1* and *srd5alpha2*) mRNA during *Silurana* (*Xenopus*) *tropicalis* metamorphosis. Real-time reverse transcriptase-polymerase chain reaction analyses revealed that all of the genes were expressed in the brain and for most of the genes expression increased during development, with the exception of *dio2*, *srd5alpha1* and *srd5alpha2*. The ability of premetamorphic tadpoles to respond to exogenous THs was used to investigate the regulation of TH- and sex steroid-related genes in the brain during development. Exposure of premetamorphic tadpoles to triiodothyronine (T3; 0, 0.5, 5 and 50 nm) for 48 h resulted in concentration-dependent increases in *trbeta*, *dio2*, *dio3*, *eralpha* and *erbeta*. Expression of *srd5alpha2* showed large increases (six- to 7.5-fold) for all three concentrations of T3. No changes were detected in *dio1*, *ar* and *cyp19* transcript levels; however, cyp19 activity increased significantly at 50 nm T3. The results obtained suggest that expression of TH-related genes and *er* during development could be regulated by rising levels of THs, as previously documented in *Lithobates* (*Rana*) *pipiens*. The positive regulation of *srd5alpha* by T3 in the brain suggests that endogenous TH levels help maintain or control the rate at which *srd5alpha* mRNA levels decrease as metamorphosis progresses. Finally, we have identified sex steroid-related genes that are responsive to T3, providing additional evidence of crosstalk between THs and sex steroids in the tadpole brain.

Amphibian metamorphosis is a complex developmental process, tightly controlled by thyroid hormones (THs) (1). The process is separated into three specific periods: premetamorphosis, prometamophosis and metamorphic climax. Premetamorphosis [Nieuwkoop and Faber (NF) stages 46–54] is the period of early tadpole growth and development characterised by low levels of circulating THs. The growth of the hind limbs and toe differentiation is accelerated during prometamorphosis (NF 55–57). This period is characterised by a rapid increase in the concentrations of endogenous THs. During metamorphic climax (NF 58–65), TH levels peak and rapid morphological changes occur, such as the emergence of the forelimbs and tail resorption (1, 2). The central nervous system (CNS) in tadpoles is an important target of THs. During metamorphosis, the CNS is extensively remodelled; for example, with re-structuring (disappearance of certain larval neuronal structures and the development of adult structures), axon guidance and growth, cell proliferation and death (3, 4).

The two THs, 3,5,3′-triiodothyronine (T3) and thyroxine (T4), mediate their physiological effects by binding to nuclear thyroid hormone receptors (tr) encoded by two genes *tralpha* and *trbeta* (5). The metabolism of THs is regulated by three types of deiodinases (type I, II and III). Deiodinase type I (dio1) catalyses outer-ring deiodination to produce T3 from T4 and also inner ring deiodination to produce reverse T3 (rT3, inactive) from T4. Type II deiodinase (dio2) exclusively activates THs by catalyzing the conversion from T4 to T3. By contrast, type III deiodinase (dio3) inactivates THs by inner-ring deiodination of T4 and T3 to produce rT3 and T2 (diiodothyronine), respectively (6). Localised activity of the different types of dio in target tissues controls local THs levels that allow the various tissues to undergo independent and differentially-timed development during metamorphosis (7). The tadpole brain has been shown to have one of the highest concentration of *tr* mRNA relative to other tissues (8) and *dio* transcripts have also been detected in the brain (9–12). Apart from being a target of THs, the CNS is also a target for sex steroids (i.e. oestrogens and androgens) that regulate brain sexual development and reproduction. For example, oestrogen is known to regulate and organise the neuroendocrine circuits and controlling reproductive functions in fish (13). In rodents, testosterone masculinises the brain via conversion to oestrogen by the enzyme aromatase (CYP19) (14) and/or by binding directly to the androgen receptor (AR) (15). Previous research in our laboratory has shown that T3 regulates the expression of the oestrogen-responsive genes: oestrogen receptor alpha (*eralpha*) and *cyp19* in the brain of *Lithobates* (*Rana*) *pipiens* (11). In addition, in *Silurana* (*Xenopus*) *tropicalis* whole body larvae, T3 increases the expression of androgen-related genes, 5α-reductase type 1 (*srd5alpha1*) and *ar* (16). Therefore, the present study aimed to investigate whether TH- and sex steroid-related genes are also regulated by T3 in the brain of *S*. *tropicalis* tadpoles.

During premetamorphosis, tadpoles have very low levels of THs but they are responsive to exogenous THs; therefore, this is an excellent developmental period to examine the functions and mechanism of action of THs (4). We used this model system to examine the effects of THs on the tadpole brain. We first established developmental profiles of TH- and sex steroid-related genes in the brain during *S. tropicalis* natural metamorphosis and adulthood. Then, premetamorphic tadpoles were exposed to T3 and transcript levels of TH-, oestrogen- and androgen-related genes and activity of cyp19 were measured in the brain of *S. tropicalis*.

## Materials and methods

### Animals

*Silurana tropicalis* frogs were reared in dechlorinated and aerated water from the University of Ottawa Animal Care facility (Ontario, Canada). Fertilised eggs were obtained from five pairs of frogs by injecting human chorionic gonadotrophin hormone (hCG; Sigma, St Louis, MO, USA) into the dorsal lymph sac of adult *S. tropicalis*. Both males and females received a priming injection of 12.5 IU of hCG followed by a boosting injection of 100 IU of hCG after 20 h. Eggs and larvae were raised in Petri dishes at 24–25 °C containing modified Ringer’s solution (0.1 m NaCl, 1.8 mm KCl, 2.0 mm CaCl_2_, 1.0 mm MgCl_2_, 300 mg/l NaHCO_3_; 1 : 9 v/v) and 0.04 mg/l of the antibiotic gentamycin (Sandoz Canada Inc., Boucherville, QC, Canada). When the tadpoles began feeding, they were transferred to 12-litre tanks containing aerated water (pH = 7.5–8.0, dissolved oxygen = 80–85%, temperature = 23–24 °C, conductivity = 850–900 μS) and fed Sera Micron (Pondside Herp Supplies, Indian Harbor Beach, FL, USA) twice a day. Staging was determined by following the NF (17) developmental table. A 12 : 12 h light/dark cycle was maintained (lights on 07.00 h). The care and treatment of animals used in this study were in accordance with the guidelines of the Animal Care Committee of the University of Ottawa and the Canadian Council on Animal Care.

### Brain collection for developmental profiles

**Fig. 1:**
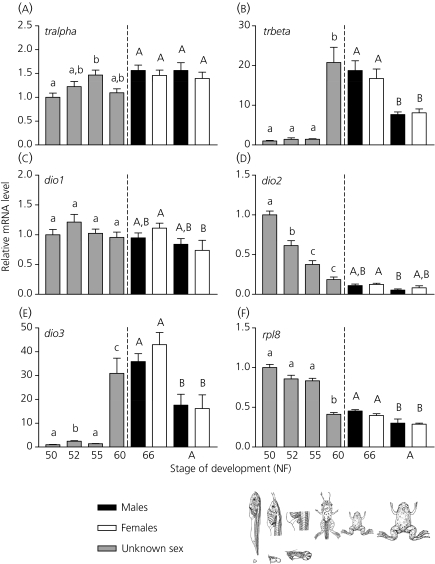
Brain developmental profiles of thyroid hormone-related genes during *Silurana tropicalis* metamorphosis and adulthood. Transcript levels of *tralpha* (a), *trbeta* (b), *dio1* (c), *dio2* (d) and *dio3* (e) were measured in whole brain from Nieuwkoop and Faber (NF) stage 50 until adulthood. Levels of mRNA are expressed relative to NF 50 and are normalised to RNA content. Results for the reference gene ribosomal protein L8 (*rpl8*; f) are also presented. Bars represent the mean ± SEM. Different letters indicate statistically significant differences between stages (n = 5–8 pools; P<0.05). Brain samples for NF 66 and adulthood were statistically analysed separately from NF 52–60 samples (for details, see Materials and methods). Main morphological characteristics (i.e. whole body and hind limb diagrams) are included for each NF stage of development. Note that the scales of the *y*-axis vary between genes. A, adult.

Samples of whole brain for gene expression were taken at different NF stages of development (Fig. 1): 50 and 52 (premetamorphosis), 55 (prometamorphosis), 60 (beginning of metamorphic climax) and 66 (juvenile). Tadpoles were anaesthetised by immersion into 1% of 3-aminobenzoic acid ethyl ester (MS-222; Sigma), euthanised by decapitation, and the brain was dissected, frozen on dry ice and kept at −80 °C. For NF 50–55, brains were pooled (two to five per pool; n = 8 pools) to ensure sufficient material for RNA isolation. For NF 60 and 66, brains were analysed individually (n = 8). In addition, to compare the gene expression during development with adult gene expression, five mature males and five mature females from our colony were sacrificed using 4% MS-222 and the brain was dissected and frozen. For the cyp19 activity assay, whole brains were also collected for NF stages 52, 55, 60, 66 following the same procedure as for the gene expression samples.

### T3 exposure

Premetamorphic tadpoles (NF stage 52–54) were exposed to three nominal concentrations of T3 (0.5, 5 and 50 nm; 3,3′,5-triiodo-L-thyronine; Sigma) or a dimethyl sulfoxide (DMSO; Sigma) solvent control for 48 h. The final DMSO concentration in the tanks was 0.005% in all treatments. The density in all the tanks was one tadpole per litre. Chemical additions were not renewed during the 48-h period. At the end of the exposure, tadpoles were anaesthetised by immersion into 1% of MS-222 and euthanised by decapitation. Brain was dissected, frozen on dry ice and kept at −80 °C.

### RNA isolation and cDNA synthesis

Total RNA for the developmental profile and T3 exposure samples was obtained from whole brain using the Qiagen RNeasy Micro kit (including the DNase treatment set) in accordance with the manufacturer’s instructions (Qiagen, Valencia, CA, USA). Individual and pooled brains were homogenised and disrupted using a MM301 Mixer Mill (Retsch, Newton, PA, USA) at 20 Hz for 3 min. Isolated RNA was resuspended in RNase free water and stored at −80 °C. Total cDNA was prepared from 1 μg (for the developmental profile) and 2 μg (for the T3 exposure) of total RNA and 200 ng random hexamer primers (Invitrogen, Carlsbad, CA, USA) using Superscript II reverse transcriptase (Invitrogen). For the developmental samples, the reverse transcriptase reaction was modified to be carried out at 42 °C for 90 min (instead of 50 min) to increase the cDNA yield. The cDNA products were diluted 20-fold prior to PCR amplification.

### Real-time reverse transcriptase-polymerase chain reaction (RT-PCR)

Gene specific primers for real-time RT-PCR were designed and optimised as previously described (18). Real-time PCR primers for additional reference genes were designed based on GenBank sequences: *gapdh* (accession no. CR760856; forward 5′-TCACTGCCACCCAGAAGAC-3′; reverse 5′-GGATGACTTTCCCAACAGC-3′; product size: 123 bp) and *18S* (accession number X04025; forward: 5′-TCAACACGGGAAACCTCAC-3′; reverse: 5′-AGACAAATCGCTCCACCAAC-3′; product size: 117 bp). Primers were designed using Primer 3 (http://fokker.wi.mit.edu/primer3/input.htm) and primer concentrations were optimised to obtain a minimum threshold cycle and a maximum change in fluorescence. The optimised primer concentration for *gapdh* was 200 nm and for *18S* it was 150 nm. The specificity of the primer sets was confirmed by cloning and sequencing the single amplicon obtained. Expression of target genes in the brain was analysed using dual-labeled fluorescent probes [for *eralpha*, *erbeta*, *cyp19* and *rpl8* (ribosomal protein L8)] and SYBR Green I [for *tralpha*, *trbeta*, *dio1*, *dio2*, *dio3*, *ar*, *srd5alpha1*, *srd5alpha2*, *actb* (β-actin), *ef1alpha*, *gapdh* and *18S*] real-time RT-PCR assays using a MX3000P real-time PCR system (Stratagene, La Jolla, CA, USA) as previously described (18).

### Data analysis

The relative standard curve method was used to interpolate relative mRNA abundance of target and reference genes within each sample. The standard curves were generated using a cDNA mix of NF 60–66 brain samples (for the developmental profiles), and using equal parts of cDNA from each treatment (for the T3 exposure). Reaction efficiencies were 90–110% with an R^2^ ≥ 0.990. Samples were run in duplicate along with a negative template control (RNase-free water instead of cDNA template) and a negative reverse transcriptase control (cDNA template for which water was added instead of Superscript II). Data for each target were averaged and normalised to RNA content (19). Data for the developmental profiles are presented as fold change relative to NF 50 and data for the T3 exposure are expressed relative to the control group.

### Aromatase activity assay

Aromatase activity in the brain during metamorphosis and after T3 exposure was measured by a radiometric method modified from Du *et al.* (20) and previously optimised for frog brain in our laboratory (18). Briefly, cyp19 activity was measured in pools of four to six brains (n = 5–6 pools) for the developmental profile and six or seven brains (n = 5 pools) for the T3 exposure. Cofactor (NADPH system) and ^3^H-androstenedione (^3^H-A) were first incubated for 30 min at 37 °C. After this pre-incubation, brain homogenates were added to a mix of cofactor and ^3^H-A, and incubated for 80 min at 25 °C. Aromatase activity was determined by the release of tritiated water from the C-1β carbon atom of 1β-^3^H-androstenedione during its conversion to oestrogen. Tritiated water was extracted twice with a charcoal solution and radioactivity was counted. Aromatase activity is expressed as fmol ^3^H_2_O/h per mg protein.

### Statistical analysis

Statistical analysis was performed using S-Plus 8.0 (Insightful Corporation, Seattle, WA, USA). Data for all the genes were first tested for normality and homogeneity of variance using the Kolmogorov–Smirnov test and Levene’s test, respectively. When the assumptions were not met, the data were transformed as required (e.g. log_10_, square root) and retested for normality and homogeneity of variance. Data were analysed by one-way anova, except for NF 66 and adult brain samples, which were analysed using a two-way anova to examine sex differences. Analyses were followed by the Bonferroni multiple comparisons test. When data failed to meet assumptions even after being transformed, the nonparametric Kruskal–Wallis test on ranks was used. P<0.05 was considered statistically significant.

## Results

### Brain developmental profiles during metamorphosis and adulthood

**Fig. 2:**
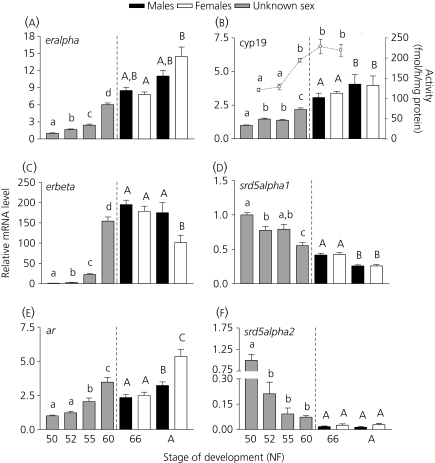
Brain developmental profiles of sex steroid-related genes during *Silurana tropicalis* metamorphosis and adulthood. Transcript levels of *eralpha* (a), cyp19 mRNA and activity (b) *erbeta* (c), *srd5alpha1* (d), *ar* (e) and *srd5alpha2* (f) were measured in whole brains from Nieuwkoop and Faber (NF) stage 50 until adulthood (*y*-axis on the left). Levels of mRNA are expressed relative to NF 50 and are normalised to RNA content. Enzyme activity for cyp19 (b) was measured from NF 52–66 (*y*-axis on the right) and is expressed in fmol/h normalised to protein content. Bars represent the mean ± SEM. Different letters indicate statistically significant differences between stages (n = 5–8 pools; P<0.05). Brain samples for NF 66 and adulthood were statistically analysed separately from NF 52 to NF 60 samples (for details, see Materials and methods). Significant differences in enzyme activity levels are indicated by small letters (n = 5–6; P<0.05). Note that the scales of the *y*-axis vary between genes. A, adult.

We established developmental profiles of TH- and sex steroid-related genes by sampling at five NF stages of development (for main morphological characteristics, see Fig. 1): 50 and 52 (premetamorphosis; foot paddle stages), 55 (prometamorphosis; hind limb development), 60 (beginning of metamorphic climax; forelimb emergence) and 66 (juvenile frog; tail completely resorbed). For all of the genes assessed, transcripts were detected throughout metamorphosis and in the adult brain (Figs 1 and 2). All the reference genes tested (*rpl8*, *ef1alpha*, *actb* and *gapdh*) changed during development; therefore, we decided to present the gene expression data normalised to RNA content only (19). Fig. 1(f) presents the brain developmental profile of *rpl8* (i.e. the reference gene that varied the least during metamorphosis). The expression profiles of the receptors and enzymes show very distinct patterns and magnitude of change. Two genes that remain relatively constant throughout larval development are *tralpha* and *dio1* (Fig. 1a, c). For *tralpha*, the only difference detected was at NF 55, which showed a higher expression relative to NF 52. For *dio1*, expression in the female brain decreases significantly after metamorphosis is complete (NF 66 versus adult brain); however, these changes are relatively minor (1.5-fold). The genes that show increases during development are *trbeta*, *dio3*, *eralpha*, *erbeta*, *ar* and *cyp19* (Figs 1 and 2). Transcript levels of *trbeta* and *dio3* are low and steady during premetamorphosis (NF 50-52) and early prometamorphosis (NF 55) and levels only increase significantly (by 30- and 20-fold respectively) at the beginning of metamorphic climax (NF 60; Fig. 1b,e). In the case of *eralpha*, *erbeta*, *ar* and *cyp19*, mRNA levels gradually increase during development (Fig. 2). We also found that cyp19 activity increases during metamorphosis, very closely following the mRNA profile (Fig. 2b). Finally, the expression of three genes (*dio2*, *sdr5alpha1* and *srd5alpha2*) decreases during development and remains low during adulthood (Figs 1d and 2d,f). Sex differences in the brain of the expression of target genes are only detected at the adult stages for *erbeta* (males had a 1.7-fold higher expression than females) and *ar* (females had a 1.7-fold higher expression than males).

### Effects of T3 on brain transcript levels of thyroid hormone- and sex steroid-related genes

**Fig. 3:**
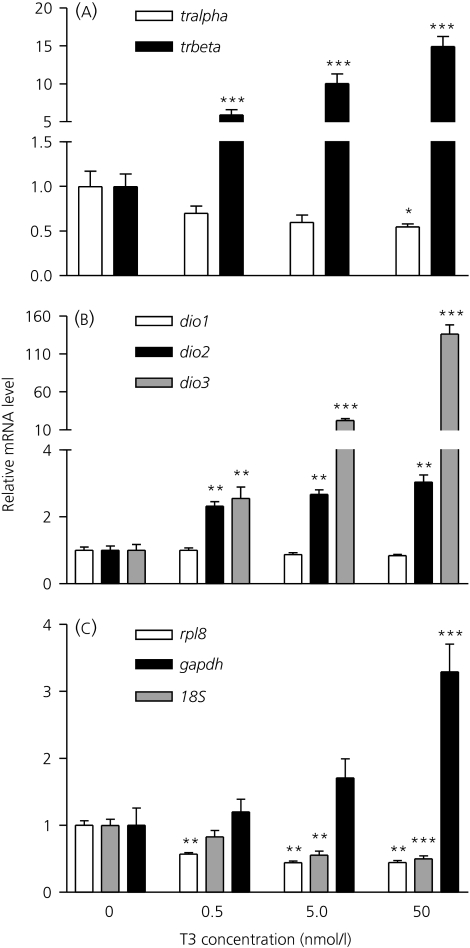
Effects of triiodothyronine (T3) exposure on the expression of thyroid hormone-related genes in *Silurana tropicalis* premetamorphic tadpoles. *Silurana tropicalis* (Nieuwkoop and Faber stage 52–54) were exposed to T3 (0, 0.5, 5, 50 nm) for 48 h. Effects of T3 on *tralpha* and *trbeta* (a), *dio1*, *dio2* and *dio3* (b) and the reference genes *rpl8*, *gapdh* and *18S* (c) are presented. Data are presented as the fold changes relative to control and are normalised to RNA content (note the broken *y*-axis for a and b). Bars represent the mean ± SEM. Asterisks represent significant differences from the control group (n = 8 pools; *P<0.05; **P<0.01; ***P<0.001). Note that the scales of the *y*-axis vary between genes.

To investigate whether T3 regulates the expression of TH- and sex steroid-related genes in the brain of *S. tropicalis*, premetamorphic tadpoles (NF 52–54) competent to respond to THs were exposed to exogenous T3 for 48 h. Treatment with T3 had no effect on mortality and 100% survivorship post-exposure was observed in all treatment groups. Exposure to T3 resulted in concentration-dependent increases in *trbeta* (5.0–15-fold; anova; P<0.001), *dio2* (2.0–3.0-fold; anova; P<0.01) and *dio3* (2.5–120-fold; anova; P<0.01) mRNA relative to control (Fig. 3). Levels of *tralpha* remained unchanged for the 0.5 and 5 nm groups but decreased significantly (2.0-fold; anova; P<0.05) at the 50 nm T3 concentration (Fig. 3a). Transcript levels for *dio1* did not change with T3 treatment (Fig. 3b). The reference genes *rpl8*, *gapdh* and *18S* changed with T3 (Fig. 3c; anova; P<0.01); therefore, the gene expression data is normalised to RNA content, as suggested by Huggett *et al.* (19), and as we have previously reported (16, 18).

**Fig. 4:**
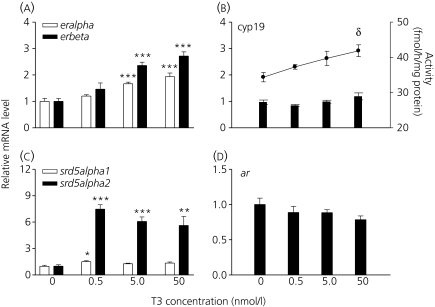
Effects of triiodothyronine (T3) exposure on the expression of sex steroid-related genes in *Silurana tropicalis* premetamorphic tadpoles. *Silurana tropicalis* (NF 52–54) were exposed to T3 (0, 0.5, 5, 50 nm) for 48 h. Effects of T3 on *eralpha* and *erbeta* (a), cyp19 mRNA and activity (b), *srd5alpha1* and *srd5alpha2* (c) and *ar* (d) are presented. Gene expression data (*y*-axis on the left) are presented as the fold changes relative to control and are normalised to RNA content. Enzyme activity (*y*-axis on the right; b) is expressed in fmol/h normalised to protein content. Bars represent the mean ± SEM. Asterisks represent significant differences in gene expression levels from the control group (n = 8 pools; *P<0.05; **P<0.01; ***P<0.001). Significant differences in enzyme activity levels are indicated by delta (δ; n = 5; P<0.01) relative to the control group. Note that the scales of the *y*-axis vary between genes.

The effects of T3 on sex steroid-related gene expression and cyp19 activity are shown in Fig. 4. Exposure to T3 resulted in concentration-dependent increases in *eralpha* (1.8–2.0-fold; anova; P<0.001) and *erbeta* (2.4–2.8-fold; anova; P<0.001) mRNA (Fig. 4a). Expression of *cyp19* was not affected by T3; however, we observed a significant increase (21% relative to control; anova; P<0.05) in cyp19 activity at the 50 nm concentration (Fig. 4b). Gene expression levels of *srd5alpha2* increased significantly (between six- and 7.5-fold; anova; P<0.01) at all concentrations of T3, whereas levels of *srd5alpha1* increased significantly at 0.5 nm T3 relative to the control group (1.5-fold; anova; P<0.05; Fig. 4c). Transcript levels of *ar* did not change after exposure to T3 (Fig. 4d).

## Discussion

In the present study, we used *S. tropicalis* tadpoles to investigate the effects of THs on brain gene expression. There is evidence that the thyroid and reproductive axes interact during tadpole development (11, 16, 18, 21). Therefore, the target genes that we chose to analyse are those related to TH, oestrogen and androgen receptors and synthesis enzymes. In the second part of the study, tadpoles were exposed to three concentrations of T3 (0.5, 5.0 and 50 nm) for 48 h. We followed the same protocol (e.g. T3 concentrations, stages of tadpoles, duration of the exposure) as in our previous study using *L. pipiens* (11) to allow valid comparisons between the two species. In the present study, we report that developmental expression and T3-regulation of TH-related genes are similar in *S. tropicalis* as in other frogs. By contrast, this is not the case for oestrogen responsive genes. Moreover, T3 can regulate expression of androgen synthesis enzymes in the brain.

### Expression of thyroid hormone receptors and deiodinases during metamorphosis

Developmental profiles of *tr* and *dio* in the brain during natural metamorphosis have been previously established in *S. tropicalis* and other frog species. In the case of *trbeta*, the developmental profile and response to T3 obtained in the present study are very similar to data from *Xenopus laevis* (22), *L. pipiens* (11), and to those previously published for *S. tropicalis* (23). In *X. laevis*, *trbeta* mRNA increases in parallel with TH levels during metamorphosis and after T3 treatment (24).

This is the first time that expression profiles have been established for all three *dio* in the brain of *S. tropicalis*. To the best of our knowledge, we also present for the first time the developmental profile of *dio1* in a frog brain. In the case of *dio2* and *dio3*, the present results are similar to the profiles in *L. pipiens* (11). Interestingly, *dio1* remained fairly constant during metamorphosis and after T3 exposure, suggesting that this enzyme does not play a major role in brain remodelling during metamorphosis. The results for *dio1* are in marked contrast to *dio2* and *dio3*, which show a very dynamic pattern of expression and T3 regulation. The positive regulation of *dio2* by T3 is surprising because we would expect *dio2* to decrease with T3 exposure (as observed in the developmental profile, *dio2* decreases at metamorphic climax when T3 levels are highest). However, this has been observed previously in whole larvae of *S. tropicalis* (16) and *X. laevis* (10) and in the brain of *L. pipiens* (11) and *S. tropicalis* tadpoles (25) exposed to T3. The molecular mechanism underlying this response needs to be investigated further. Increases in *dio3* mRNA during development and after T3 exposure are also consistent with previous studies (10, 11, 16) and can be explained by the presence of a TH-responsive element in the promoter region of the *X. laevis* and *Rana catesbeiana dio3* genes (26, 27). Low levels of *dio2* and high levels of *dio3* during metamorphic climax indicate that the brain has reduced T3 synthesis but has started inactivating THs to protect itself from high levels of circulating THs because developmental remodelling of the brain has been completed (9).

### Expression of oestrogen- and androgen-related genes during metamorphosis

Our treatments were effective and the T3 responses in TH-related genes in *S. tropicalis* brain were highly comparable to several other species. The second question that we addressed in the present study was whether T3 could affect the expression of sex steroid-related genes in the brain during metamorphosis. We measured for the first time oestrogen- and androgen-related genes in the brain of *S. tropicalis* during metamorphosis. Although both *eralpha* and *erbeta* increase during development in the brain, the magnitude of change of their mRNA levels is very different. Of all the genes analysed in the present study, *erbeta* showed the highest increase (150-fold) at NF 60, whereas *eralpha* showed a more moderate increase (six-fold). Comparing the developmental profiles to the T3 exposure results helps elucidate whether endogenous THs are important regulators of gene expression. Interestingly, we found that both *er* mRNAs increased at the beginning of metamorphic climax and they were also positively regulated by T3. However, the relative increases were not as dramatic in the short T3 exposure compared to the metamorphic peak). The expression of *er* mRNAs has been shown to be regulated by oestrogen and oestrogenic compounds in frog and fish brain (28–30). We suggest that part of the increase in *er* mRNAs during development could be a result of rising levels of THs during metamorphosis and the other part could be a result of rising levels of oestrogen in the brain via increasing cyp19 activity during development (present study). This study with *S. tropicalis* confirms the previous findings in the brain of *L. pipiens* where *eralpha* also increased during metamorphosis and after T3 exposure (11). In the adult brain, we found that *erbeta* mRNA was higher in males than females, implying that there may be sex differences in the T3 regulation of *er* mRNA in the tadpole brain. However, this idea and the molecular mechanism by which T3 induces *er* expression need to be investigated further.

We found that both cyp19 mRNA and activity increase in the brain during *S. tropicalis* metamorphosis and this is similar to gene expression profiles in *X. laevis* (31) and *L. pipiens* (11). T3 did not affect *cyp19* mRNA, which is in marked contrast with results in *L. pipiens* where *cyp19* mRNA decreased with T3 concentration (11). This difference between the two studies suggests there might be species differences in T3 regulation of *cyp19*; however, additional studies using other frog species will help further elucidate *cyp19* regulation in the frog brain. Furthermore, we found that T3 increases cyp19 activity in the brain of *S. tropicalis*. To our knowledge, this is the first time that an increase in cyp19 activity has been reported after T3 treatment in the frog brain. In mammals, most of the research has focused on the effects of TH on gonadal development and function. Exposure to TH decreases gonadal CYP19 activity *in vitro* in pigs (32) rats (33, 34) and mice (35). Therefore, the molecular mechanism and physiological consequences of T3 regulation of cyp19 activity in the brain are unknown at this point. One way that T3 could affect cyp19 activity without affecting mRNA level is by regulating the enzyme at the post-translational level (e.g. phosphorylation of the enzyme) (36) or by another indirect mechanism.

The profiles of *srd5alpha1* and *srd5alpha2* differ significantly with respect to the profiles of the other sex steroid-related genes. The expression of the two enzymes involved in the processing of testosterone for 5α-dihydrotestosterone (5α-DHT) synthesis decreases during development and remains low during adulthood in the brain. Urbatzka *et al.* (31) measured *srd5alpha1* and *srd5alpha2* mRNAs by semi-quantitative RT-PCR in the brain of *X. laevis* and, in general, their profiles are very similar to the ones reported in the present study for *S. tropicalis*. Immunohistochemical analyses have also shown that srd5alpha1 is present in the brain of *Rana esculenta* tadpoles during metamorphosis (37). Exposure to all three concentrations of T3 resulted in a large increase (six- to 7.5-fold) in *srd5alpha2* but only a small increase (1.5-fold) in *srd5alpha1*. Previous studies have found that T3 induces *srd5alpha1* mRNA in whole larvae of *S. tropicalis* (16) and, in rats, hypothyroidism causes decreases in hepatic Srd5alpha expression and activity, an effect that was restored with T4 treatment (38). Srd5alpha2 is also involved in the conversion of progesterone into 5α-reduced metabolites such as allopregnanolone. Allopregnanolone has been shown to be involved in neurogenesis, regulating cell death and proliferation *in vitro* in rat and human neuronal stem cells (39) as well as in the developing sheep brain (40). TH promotes neurogenesis in the spinal cord of *X. laevis* (41), and is involved in neuronal proliferation and migration during vertebrate development (42). Taken together, the induction of *srd5alpha1* and *srd5alpha2* by T3 could be related to the roles of TH and neurosteroids in brain development. The comparison of developmental expression profiles and the results obtained after T3 exposure of *srd5alpha1* and *srd5alpha2* reveal marked differences. During metamorphosis, *srd5alpha1* and *srd5alpha2* decrease in the brain, whereas they increase following T3 exposure. These results indicate that rising endogenous THs are not directly responsible for developmental decreases in *srd5alpha* expression in the tadpole brain. Whole body measurement of testosterone and 5α-DHT indicates that androgens decrease during *X. laevis* development (43). In *S. tropicalis* larvae exposed to finasteride (a 5α- and 5β-DHT synthesis inhibitor), we have observed a decrease in *srd5alpha2* (18, 21). In rats, 5α-DHT can induce *Srd5alpha* in the prostate (44) and *Srd5alpha2* in the adult brain (45). These studies suggest that expression of *srd5alpha* can be regulated by its own enzymatic reaction product (i.e. feedforward control of 5α-DHT on *srd5alpha*). Therefore, the decrease in *srd5alpha* mRNA during development observed in the present study could be linked to the decrease in 5α-DHT levels. On the other hand, rising TH levels likely counteract this to maintain or control the rate at which *srd5alpha* mRNA levels decrease as metamorphosis progresses. Another explanation involves the action of oestrogens. Oestradiol has been shown to inhibit Srd5alpha activity in rat skin (46) and adrenal tissue (47). In addition, in *S. tropicalis*, exposure to the aromatase inhibitor fadrozole increases hepatic *srd5alpha1* and *srd5alpha2* mRNA levels (21) and, in fish, fadrozole increases circulating androgen levels (48). These studies suggest that oestrogen can inhibit androgen synthesis in the tadpole as in adult frogs (49). In the present study, we found that brain cyp19 activity increases during *S. tropicalis* metamorphosis; therefore, this putative increase in oestrogen could inhibit srd5alpha activity in the brain, leading to a decrease in 5α-DHT and *srd5alpha* mRNA as a result of feedforward control. Future experiments are needed to test these hypotheses of the complex interplay between THs, androgens and oestrogens, as well as the physiological consequences of the regulation of *sr5alpha* by T3.

Taken together, the present results indicate that TH-related genes display very similar developmental profiles and T3 responses in the brain of different frog species. We found that the brain is an important site of TH regulation of not only TH-responsive genes, but also sex steroid receptor and enzyme genes. In the case of sex steroid-related genes, T3 regulation of *eralpha* appears to be common to both *S. tropicalis* and *L. pipiens*; however, this is not the case for *cyp19* and additional research is needed for the androgen-related genes. Finally, we present supporting evidence of a crosstalk between TH and sex steroids in the developing brain of *S. tropicalis*. The present study provides an important baseline to determine the physiological consequences of this interaction during the remodelling of the frog brain.
